# 1609. Engagement in Care, Awareness, and Interest in Long-Acting Injectable Anti-Retroviral Therapy

**DOI:** 10.1093/ofid/ofad500.1444

**Published:** 2023-11-27

**Authors:** Jacob Stout, Maxwell Allamong, Frances Hung, Katherine Link, Cliburn Chan, Charles Muiruri, John Sauceda, Mehri McKellar

**Affiliations:** Duke University School of Medicine, Durham, North Carolina; Duke Institute for Survey Methodology, Durham, North Carolina; Duke University, Durham, North Carolina; Duke University, Durham, North Carolina; Duke University, Durham, North Carolina; Duke University, Durham, North Carolina; University of California San Francisco, San Francisco, California; Duke University Hospital, DURHAM, North Carolina

## Abstract

**Background:**

Long Acting Injectable (LAI) therapy to treat HIV is now a viable alternative to daily oral medications. The success of early roll-out of LAI to eligible patients requires a better understanding of patients’ awareness and interest in this novel therapy.

**Methods:**

We administered an electronic survey to patients attending an urban HIV clinic in the US South (Duke University). Eligible participants were 18+ years old with a most recent HIV-1 viral load < 200 copies/ml, without any evidence of genotypic resistance to LAI components or chronic hepatitis B. Survey recipients were asked questions on their current treatment, their awareness and interest in LAI, and were asked to rate common benefits and concerns associated with LAI. After probing awareness of LAI, patients were provided further details on LAI for consideration for the rest of the survey. The survey also included the 3-item Brief Index of Engagement in HIV Care, a patient-reported measure of engagement in HIV care that has predicted future retention in care outcomes.

**Results:**

Between January-April 2023, 480 patients were screened; 319 were found to be eligible, and 159 (50%) completed the survey. 73% were male; 44% Black, 7% Latinx; mean age was 49. The majority (119, 76%) were aware of injectable treatments, and 87 (56%) were interested in LAI. Among proposed benefits of injectables, ease of travel without pills, fewer medication interactions, and lack of daily pill-taking were most appealing. Among proposed concerns with injectables, out of pocket cost and insurance coverage of the new medicine were most worrisome. Black patients were more likely to be interested in LAI, and interested patients tended to be younger, single, and more recently diagnosed with HIV. Overall, patients surveyed were highly engaged in care (Mean 4.5/5, SD: 0.5)., but engagement was not predictive of awareness or interest in LAI.

**Figure:**
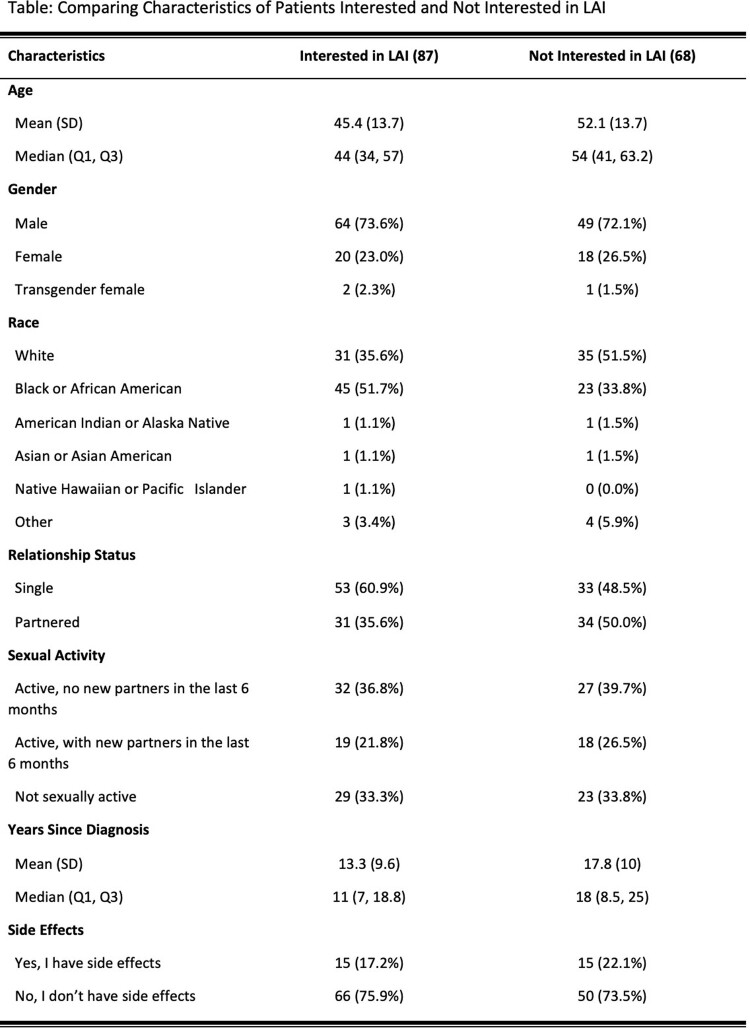

This table summarizes and compares the key patient characteristics between the patients who rated themselves interested or not interested in LAI

**Conclusion:**

As we roll out LAI in real world settings, it will be helpful to characterize which patients are most interested in order to optimize uptake and benefit. Ease of travel without pills, not having to remember daily doses, and fewer medication interactions were the most appealing reasons, while concerns about both insurance coverage and out of pocket cost will need to be addressed.

**Disclosures:**

**Mehri McKellar, MD**, Gilead Sciences, Inc: Grant/Research Support

